# Early Childhood Caries and Oral Health-Related Quality of Life: Evaluation of the Effectiveness of Single-Session Therapy Under General Anesthesia

**DOI:** 10.1055/s-0042-1757210

**Published:** 2022-10-28

**Authors:** Francesco Saverio Ludovichetti, Andrea Zuccon, Donatella Cantatore, Giulia Zambon, Luca Girotto, Patrizia Lucchi, Edoardo Stellini, Sergio Mazzoleni

**Affiliations:** 1 Dentistry Section, Department of Neurosciences, , Università degli Studi di Padova, Padova, Italy; 2 Unità Operativa di Chirurgia Orale e Odontostomatologia, Ospedale S. Lorenzo, Trento, Italy; 3 Dentistry Section, Department of Neurosciences, , Università degli Studi di Milano, Milano, Italy

**Keywords:** ECC, dental care, general anesthesia, ECOHIS

## Abstract

**Objective** The aim of this study is to evaluate whether the treatment of ECC, performed in a single-session dental treatment under general anesthesia, can affect the quality of life of pediatric patients. It was assessed whether risks and discomforts involved in SSGA are outweighed by its effectiveness and reliability in improving oral health-related quality of life.

**Materials and Methods** The quality of life that was assessed in this prospective study was oral health-related quality of life (OHRQL). Pediatric patients aged between 3 and 6 years with ECC undergoing dental treatment in SSGA were asked to fill in the Early Childhood Oral Health Impact Scale (ECOHIS) form both before and 1 month after the intervention. The data obtained were then statistically elaborated and analyzed to evaluate the actual significance of the differences found between the values before and after treatment and between the two sexes.

**Results** Mean ECOHIS score before treatment was 30.58, following a large decrease after treatment, with a mean score of 2.94. Most parameters show a significant improvement between pre- and post-SSGA treatments, mainly those related to oral–dental pain, daytime irritability, and impact on family environment. Average ECOHIS scores for males and females are 31.72 and 29.76 before treatment and 3.55 and 2.52 1 month after treatment, respectively, showing no statistically significant differences.

**Conclusion** The dental treatment of young children under SSGA is associated with considerable improvement in their OHRQL. It can be considered an effective and reliable way of managing cases that cannot be dealt with by alternative methods.

## Introduction

In preschoolers between 3 and 6 years of age, the incidence of early childhood caries (ECC) represents one of major oral health problems. This is due both to the difficulty of clinical and therapeutic management of the young patient (because of his poor compliance) and to the serious esthetic, functional, and general health consequences, which affect the child's psychophysical development. 1 2 The etiology of ECC is complex and multifactorial: it is the result of incorrect behavioral habits associated with predisposing factors that severely impair the quality of life of young patients. 3 4 It has been defined as “the presence of one or more decayed (noncavitated or cavitated lesions), missing (due to caries), or filled tooth surfaces in any primary tooth in a child under the age of 6 years.” 5 ECC can lead to pain and dental emergencies, recurrent infections, malocclusion, development of new caries in mixed dentition, possible alterations in development and growth, as well as future dental anxiety and phobia as adults. 6 The first manifestation is usually hypersensitivity to heat and cold, acids and sugars; then pain on mastication appears. The child may reduce the intake of food and the parents compensate by giving fermentable carbohydrates which make the situation worse. Untreated carious lesion deepens over time leading to pulpitis with intense, spontaneous, and lasting pain; the condition may evolve into an apical abscess with cheek tumefaction, often associated with fever and adenopathy. The pain occurs mainly at night, with long-lasting episodes of hypalgia, making the child irritable, nervous, and inattentive at school during the day. This situation has repercussions on whole family context, both emotionally and economically. 7 8 Another consequence of ECC is orthodontic damage, due to early loss of deciduous teeth, with subsequent malocclusion of the dental arches and possible defects in swallowing and phonation, as well as psychological outcomes. 8 Considering the young age of the patients concerned and the severe consequences, the World Health Organization prescribes to use different strategies in the management of ECC. The aim is preventing or reducing the progression of carious lesions, as well as making the parent more aware of erroneous behavior and seeking sufficient cooperation for outpatient clinical treatment. 9

When the child is cooperative, many treatment options are available, from standard restorative techniques to the use of preformed crowns, depending on the amount of tooth destruction. 10

However, if the child is uncooperative and it is not possible to safely perform dental procedures in the outpatient clinic, but dental treatment is still essential and cannot be postponed, a more invasive therapeutic approach is used: single session under general anesthesia (SSGA) treatment, which represents the only curative option capable of ensuring adequate quality and duration in time. 11 However, all anesthetic agents present potential risks to the general health of the patient in terms of both morbidity and mortality, so their use should be limited to situations where routine therapies cannot be used. 12

The aim of the study is to find out whether and to what extent treatment of ECC with SSGA method can affect the quality of life of the pediatric patients involved, through a prospective statistical evaluation of questionnaires filled in by the families of young patients.

## Materials and Methods

The study participants were children of either gender visiting Department of Pediatric Dentistry, Faculty of Dentistry, Università di Padova (Italy), between January 2011 and December 2016. Written consent from parent(s) was obtained prior to oral examination and interview. Ethical approval was waived by the ethic committee.

### Eligibility Criteria

The study group consisted of preschooler patients in need of dental treatment in SSGA. This group was made up of subjects aged between 3 and 6 years with ECC (Decayed, Missing, Filled Teeth (DMFT) more than 8) (whose requirements were included in the national guidelines for general anesthesia [GA]) for whom recourse to SSGA was assessed as absolutely essential (cases of acute infection or dentoalveolar abscesses where drug therapy or drainage procedures by other methods were inadequate or unsuccessful; uncooperative patients in whom it would not be possible to carry out treatment safely). Age group between 3 and 6 years is the group of patients most frequently in need of SSGA, considering the close relationship between personality development and age.

**Fig. 1:**
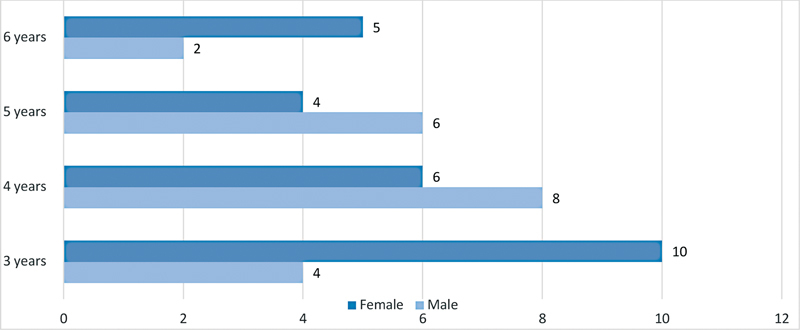
Patient distribution by age and gender.

Total 45 patients were involved, 20 males and 25 females. The average age was 4.23 years, 4.3 for males and 4.2 for females ( Fig. 1 ).

### Questionnaires

Patient's parents filled two different questionnaires in different moments: a first questionnaire was given to the patient's parent(s) on the day of SSGA. It was related to the child's oral state within the past 3 months. A second questionnaire, relating to child's oral condition since the treatment under SSGA, was given to the participants 1 month after their SSGA, during their scheduled postoperative review appointment. Both consisted of the Early Childhood Oral Health Impact Scale (ECOHIS) form.

### Early Childhood Oral Health Impact Scale

Oral health-related quality of life (OHRQL) was assessed. The OHRQL measurement instrument used was the “Early Childhood Oral Health Impact Scale” (ECOHIS). 13 It is made up of 13 parameters for each of which a score from 0 to 4 is given, considering the answers according to the frequency of the event: never (0), almost never (1), occasionally (2), often (3), and very often (4). First nine parameters (child impact section [CIS]) refer to signs/symptoms manifested by the patient, and other four (family impact section [FIS]) concern the repercussions of the pathology on the family in its totality. In general, the ideal score, corresponding to an optimal level of the OHRQL, would be “0.” The maximum score, corresponding to the worst condition of the OHRQL, would be “52” (with a value of “36” for the part referring to the patient—CIS and “16” for the part concerning the family nucleus—FIS). 14 Considering the patients' young age, data for CIS were collected by a single trained doctor at the end of the postoperative schedule.

### Statistical Analysis

Data were submitted to Student's *t* -test for paired data to determine significant differences ( *p* = 0.05). Statistical analysis was performed using a statistical software program (IBM SPSS Statistics v22.0; IBM Corp).

## Results

Data are presented in Fig. 1 and Tables 1 to 3 .

**Table 1:** ECOHIS values

Scale	Pretreatment	Posttreatment	Difference (%)
**Child impact section**	**20.44**	**2.24**	**89**
Does the child have teeth/mouth pain?	3.31	0	100
Does the child have difficulty in taking hot/cold drinks?	3.48	0.48	86
Does the child have difficulty in taking food?	3.28	0.44	87
Does the child have difficulty in pronouncing some words?	0.78	0.04	95
Does the child miss school/kindergarten days?	2.42	0	100
Does the child find it difficult to rest?	3.28	0.48	85
Does the child seem to be irritable?	3.28	0.8	76
Does the child avoid laughing/smiling when surrounded by other children?	0.35	0	100
Does the child avoid talking when surrounded by other children?	0.26	0	100
**Family impact section**	**10.14**	**0.7**	**93**
Does the family feel responsible for the child's dental problems?	3.48	0.26	93
Does the family feel guilty for the child's oral–dental situation?	2.22	0.13	94
Has anyone in the family had to ask for days off from work?	2.2	0.09	96
Did the child's oral–dental situation require treatment that had a financial impact on the family?	2.24	0.22	90
Total	30.58	2.94	90

Abbreviation: ECOHIS, Early Childhood Oral Health Impact Scale.

**Table 2:** ECOHIS values for “male” subgroup

Scale	Pretreatment	Posttreatment	Difference (%)
Child impact section	21.3	2.65	87.56
Does the child have teeth/mouth pain?	3.4	0	100
Does the child have difficulty in taking hot/cold drinks?	3.7	0.4	89.19
Does the child have difficulty in taking food?	3.5	0.55	84
Does the child have difficulty in pronouncing some words?	0.8	0.05	93.75
Does the child miss school/kindergarten days?	2.4	0	100
Does the child find it difficult to rest?	3.3	0.65	80.3
Does the child seem to be irritable?	3.5	0.1	71.43
Does the child avoid laughing/smiling when surrounded by other children?	0.45	0	100
Does the child avoid talking when surrounded by other children?	0.25	0	100
**Family impact section**	**10.42**	**0.9**	**91.36**
Does the family feel responsible for the child's dental problems?	3.55	0.3	91.55
Does the family feel guilty for the child's oral–dental situation?	2.32	0.2	91.38
Has anyone in the family had to ask for days off from work?	2.4	0.2	91.67
Did the child's oral–dental situation require treatment that had a financial impact on the family?	2.15	0.2	90.7
Total	31.72	3.55	88.8

Abbreviation: ECOHIS, Early Childhood Oral Health Impact Scale.

**Table 3:** ECOHIS values for “female” subgroup

Scale	Pretreatment	Posttreatment	Difference (%)
Child impact section	19.94	1.96	90.1
Does the child have teeth/mouth pain?	3.24	0	100
Does the child have difficulty in taking hot/cold drinks?	3.32	0.56	83.14
Does the child have difficulty in taking food?	3.12	0.36	88.46
Does the child have difficulty in pronouncing some words?	0.76	0.04	94.74
Does the child miss school/kindergarten days?	2.44	0	100
Does the child find it difficult to rest?	3.28	0.36	89.03
Does the child seem to be irritable?	3.12	0.64	79.49
Does the child avoid laughing/smiling when surrounded by other children?	0.28	0	100
Does the child avoid talking when surrounded by other children?	0.28	0	100
**Family impact section**	**9.92**	**0.56**	**94.36**
Does the family feel responsible for the child's dental problems?	3.44	0.24	93.02
Does the family feel guilty for the child's oral–dental situation?	2.12	0.08	96.23
Has anyone in the family had to ask for days off from work?	2.04	0.09	100
Did the child's oral–dental situation require treatment that had a financial impact on the family?	2.32	0.24	89.66
Total	29.76	2.52	91.53

Abbreviation: ECOHIS, Early Childhood Oral Health Impact Scale.

Before treatment, parameters with the highest value were those related to oral–dental pain (including when eating or drinking), daytime irritability, and troubled sleep. Lowest scores were related to relational behaviors such as avoidance of smiling and speech difficulties. Mean ECOHIS score after treatment (2.94) is lower than pretreatment one (30.58), with a statistical significance difference ( *p* < 0.001 with Student's *t* -test). Average ECOHIS scores for males and females ( Table 1 ) are 31.72 and 29.76 before treatment and 3.55 and 2.52 1 month after treatment, respectively, showing no statistically significant differences ( *p* > 0.05). Most parameters, individually taken, ( Tables 1 , 2 ) show a significant improvement between pre- and post-SSGA treatments.

All the four parameters concerning the impact of the deteriorated OHRQL on family environment obtain a radical lowering of the posttreatment score, both on psychological and economic-occupational aspect. The family sense of responsibility for the child's situation improved by 92.5% and parental guilt by as much as 94.1%, while for economic-occupational aspect, the improvement (95.9 and 90.2%) was also significant.

## Discussion

First proposed by Pahel et al, 14 ECOHIS has proven its validity and reliability in preschoolers over years and is now considered the main and most qualified method for assessing OHRQL in these patients. 15 16 17 High mean value of pretreatment ECOHIS (30.58), as evidenced by this study, clearly indicates how OHRQL can be influenced by ECC. Oral pain, resulting difficulty in eating and drinking properly, and sleep disorders resulting in daytime irritability, all contribute to the OHRQL deterioration. preschoolers with ECC do not necessarily complain about pain, but rather manifest pain effects in the form of changes in their eating and sleeping habits. 18 As evidenced by many studies, 19 20 SSAG treatment of ECCs has an immediate positive effect on OHRQL, mainly in pain condition, followed by improving in the ability to eat and sleep. Parents themselves perceive an improvement in their children's quality of life. 21 In addition, it has been observed that preschoolers with severe ECC show changes in body growth. With advancing age and, presumably, increasing severity of ECC, there is a slowdown in weight gain such that older children with ECC are more likely to have a weight index in percentiles below normal ranges. It has been reported that a growth recovery phenomenon occurs following SSGA treatment of ECC. 22 23 24

In discordance with other authors, 25 26 27 the present study did not find significant differences in factors such as reluctance to smile, pronunciation difficulties, and tendency to avoid speaking. This probably comes from the age group considered (3–6 years), in which the child's self-image and acceptance by the peer group are not significantly influenced by oral health. The questionnaire involves parent(s) because children younger than 6 years are not yet able to contextualize how oral health influences their habits and therefore the quality of their daily life and their families. 23

The present study also shows how SSGA treatment has a positive effect on the FIS parameters, with important and statistically significant variations, regarding psychological condition and economic and occupational implications for families.

In accordance with previous studies, 18 19 ^,^ 28 no sex-related differences emerged, neither in initial assessment of OHRQL nor in the results achieved on it by SSGA treatment. This can probably be related to the age of the sample: preschoolers, being in prepubertal phase, present a condition of psychological development which is still substantially similar for males and females and this determines substantially superimposable responses in the face of similar oral–dental pathological pictures.

The present study demonstrates the effectiveness of the single-session treatment in SSGA in drastically improving the OHRQL of preschoolers suffering from ECC. This finding stands out, according to the literature, from inferior results obtained with other behavioral control methods used to treat ECC in pediatric patients aged 3 to 6 years. These mainly consist of fractionated treatments in several sessions with a conscious patient. 18 29 30 The explanation is linked to the optimal situation that is created for the clinician: patient is intubated and monitored by anesthesiology team, totally passive and able to tolerate any intraoral procedure, with adequate time available. This leads to deal with and solve definitively and completely problems posed by ECC, which would otherwise be difficult to achieve with similar patients treated in a conscious state. Furthermore, although it has been suggested that there may be neurotoxicity related to the anesthetic used for AG in pediatric patients, no confirmation has yet been found in humans. 31

One limitations of this study could be the small sample size taken into consideration due to unavailability of resources; considering the particular condition of this treatment, a greater sample population would be helpful, but difficult to find, to confirm the findings of our study. Moreover, the presence of other affecting factor of quality of life could have affected our results.

## Conclusion

Overall, the present study suggests that GA for dental treatment of pediatric patients aged 3 to 6 years with ECC can effectively and rapidly restore parameters indicative of oral health-related quality of life. It remains open and opportune to investigate in the same way the return, in terms of “effectiveness and reliability,” of the same dental therapies provided according to a treatment plan with repeated appointments with conscious patients.
